# Impact of a Digital Leakage Notification System on Leakage, Quality of Life, Healthcare Resource Utilisation, and Work Productivity: Interim Results from a Longitudinal Real-World Study in the UK

**DOI:** 10.3390/jcm15020663

**Published:** 2026-01-14

**Authors:** Martin Vestergaard, Amanda Gunning, Rebecca Mather, Helle Doré Hansen, Teresa Adeltoft Ajslev

**Affiliations:** 1 Coloplast A/S, Holtedam 1, 3050 Humlebæk, Denmark; dkmves@coloplast.com (M.V.); dkhdh@coloplast.com (H.D.H.); 2 Royal Devon and Exeter Healthcare NHS Trust, Exeter EX2 5DW, UK; amandagunning@nhs.net; 3 Coloplast Ltd., Nene Hall, Peterborough Business Park, Peterborough PE2 6FX, UK; gbrhc@coloplast.com

**Keywords:** ileostomy, colostomy, digital leakage notification system (Heylo™), quality of life, healthcare resource utilisation, work productivity and activity impairment

## Abstract

**Background:** Leakage is a major concern for individuals living with a stoma and may negatively impact quality of life (QoL). A digital leakage notification system (DLNS) recently launched in the UK provides timely notifications to users via their smartphone when faeces is detected underneath the baseplate. This provides predictability and enables users to take proactive measures to help avoid leakages outside the baseplate. **Methods:** A single-arm, observational, longitudinal study of the DLNS, including its associated support service, has been initiated to follow 300 users for a year in the UK to evaluate long-term health benefits of the DLNS and its implications for healthcare resource utilisation in a real-world setting. The DLNS is prescribed by healthcare professionals (HCPs), and all users were invited to participate in the study. Study participants complete questionnaires capturing data on QoL (using the Ostomy Leak Impact tool), number of leakages outside the baseplate, utilisation of ostomy products, interactions with HCPs, and work productivity (using the Work Productivity and Activity Impairment questionnaire) at baseline and then every third month for one year. Data from the planned interim analysis of the first 100 participants who had been in the study for 6 months is presented. **Results:** Use of the DLNS for 6 months together with the associated support service was associated with a 51% reduction in leakage episodes outside the baseplate (*p* < 0.001) and great improvements in QoL (*p* < 0.001). Use of the DLNS reduced the number of unplanned baseplate changes due to worry about leakage by 47% (*p* < 0.001) and overall was associated with a reduction in the number of baseplates used by 14% (*p* = 0.002). Total time spent with HCPs related to stoma care was reduced by 65% after 6 months compared with baseline (*p* < 0.001). Work absenteeism and presenteeism improved significantly with the use of the DLNS. **Conclusions:** The interim results of this prospective, longitudinal study provided first insights into the long-term benefits of the DLNS in a real-world setting. **ClinicalTrials.gov ID:** NCT06554015.

## 1. Introduction

Surgery leading to stoma formation is a life-changing intervention for the patient, which necessitates adjustments to a new life with a stoma and the establishment of new care routines [1]. Many people living with a stoma report that they struggle with self-care problems even several years post-surgery, including complications with leakage and peristomal skin, and a considerable period of time spent on stoma care every day [2,3]. An important aspect of stoma care is to identify an appropriate stoma bag and baseplate combination that ensures a secure seal between the baseplate and peristomal skin to help reduce the risk of faeces seeping underneath the baseplate [4]. Stoma care nurses (SCNs) have identified complex peristomal body profiles and incorrect product usage as important risk factors for leakage [5]. Soiling incidents can have profound and long-lasting negative implications on quality of life (QoL) for the individual, which may contribute to social isolation and negatively affect the ability to work [6].

A digital leakage notification system (DLNS), named Heylo™ (Coloplast A/S, Denmark), was recently launched in the United Kingdom (UK) to help people struggling physically and psychologically with leakage. The DLNS provides timely notifications to users via their smartphone when faeces is detected underneath the baseplate. This enables users to take proactive measures to help avoid soiling incidents. The DLNS consists of a sensor layer with two circular leakage-sensor rings; the inner ring detects leakage underneath the baseplate closest to the stoma, whilst the outer sensor ring detects if leakage is spreading. The condition of the sensors is displayed on a bespoke app on the user’s smartphone via a transmitter attached to the sensor layer [7,8]. Previous clinical studies with the DLNS, including a randomised-controlled trial, showed that its use was associated with fewer leakages outside the baseplate, less worry about leakage, better sleep, and overall improvements to QoL [7,8,9,10]. The intervention periods with the DLNS in the clinical studies ranged from 3 weeks to 3 months [7,8,9].

In the present observational study, approximately 300 DLNS users are observed over a 12-month period to investigate long-term impacts on clinical outcomes, healthcare resource utilisation, and work productivity when using the DLNS together with a remote support service in a real-world setting. The remote support service enables those using the DLNS to receive information concerning appropriate stoma care routines, leakage-related issues, and technical support on the system. The DLNS can also work as a stand-alone solution [9].

In this report, data from the planned interim analysis of the first 100 enrolled participants who had been in the study for 6 months are presented.

## 2. Methods

### 2.1. Study Design

This single-arm, open-label, observational study with a pre–post, within-subject design consisted of a baseline measurement and a 12-month observational period of people’s use of a DLNS (Heylo™; Coloplast A/S, Humlebæk, Denmark). People were invited to enrol in the study if they had been using Heylo for less than 7 days. Participants completed a questionnaire at baseline (V1) and again after 3 (V2), 6 (V3), 9 (V4), and 12 (V5) months, comparing Standard of Care (baseline) with Standard of Care + DLNS (including a remote support service). The study is conducted in the UK and intends to enrol a total of 300 DLNS users. Data from the planned interim analysis of the first 100 participants who had been in the study for 6 months are presented in this report. The study is registered on ClinicalTrials.gov: NCT06554015.

### 2.2. Enrolment of Participants

The DLNS is prescribed via healthcare professionals (HCPs) who use the Leakage Impact Assessment (LIA) tool to assess the degree to which individuals with a stoma struggle with leakage-related complications [11].

Upon receiving the DLNS and completing their registration through the bespoke Heylo™ app (Vers. 3 and 4; Coloplast A/S, Humlebæk, Denmark), all users were sent an email containing brief information about the study and a link to a webpage with additional study details. The webpage included a self-screening questionnaire where the user could actively consider eligibility criteria for participation in the study. Inclusion criteria identified those who were at least 18 years old with an ileostomy or a colostomy and who had been using the DLNS for less than 7 days. Individuals with a planned stoma reversal within 6 months of enrolment were excluded from participation in the study. Eligible users were formally enrolled in the study when they provided informed consent electronically. The distribution of study questionnaires was performed by QC Medica (Liverpool, UK). Approximately 39% of all individuals who have been prescribed the DLNS signed up for the study.

### 2.3. Patient Demographics and Endpoints

Patient demographics and pertinent clinical data (sex, age, stoma type, time since stoma surgery, reason for stoma surgery, and stoma care product characteristics) were captured via a self-reported online questionnaire. The present report includes the primary and secondary endpoints, and a selection of exploratory endpoints.

Clinical outcomes included the following:

- The primary endpoint of the study was the Emotional Impact domain score of the validated Ostomy Leak Impact (OLI) tool [12]. The two other domains of the tool (the Usual and Social Activities domain and the Coping and in Control domain) were included as explorative endpoints. The entire tool consists of 22 questions, which summarize the burden of stoma leakage. Each domain sums into a score ranging from 0 to 100, where higher scores reflect better leakage-related QoL (i.e., lower impact of stoma-leakages on different aspects of life).
- Participants reported on the number of leakages outside the baseplate (e.g., onto clothes or bed sheets) within the preceding 2 weeks.

Multiple endpoints were captured relating to healthcare resource utilisation and the ability to work:

- Recall of the number of baseplate changes (or entire system for 1-pc users) in the preceding 14 days.
- Recall of the number of baseplate changes (or entire system for 1-pc users) due to leakage worry (not due to an actual leakage) in the preceding 14 days (Secondary endpoint).
- Recall of the use of 15 different supporting products in the preceding 14 days.
- Participants reported on their usage of the DLNS with the preceding 10 baseplate changes.
- Time spent with different types of HCPs in the preceding 3 months was assessed with users reporting on the number of visits with the HCP and the duration of each visit. Participants could also provide free-text options, which are not presented in this report. Total time spent with HCPs (excluding Coloplast interactions) is estimated.
- The Work Productivity and Activity Impairment (WPAI) questionnaire was used to assess the impact of the patients’ health problems on their ability to work and to perform regular activities [13]. Health problems were defined as any physical or emotional symptom, and not specifically related to their stoma, as the questionnaire has not been validated in this patient population. The WPAI questionnaire includes four metrics, with each metric score ranging from 0 to 100. Scores reflect higher implications of health problems on the ability to work:
  ○ WPAI 1: Absenteeism (the percentage of work time missed due to health problems).
  ○ WPAI 2: Presenteeism (the percentage of impairment while working due to a health problem).
  ○ WPAI 3: Productivity loss rate (the percentage of overall work impairment due to health problems, considering both absenteeism and presenteeism).
  ○ WPAI 4: Percent activity impairment due to health problem (the percentage of daily activities affected by health problems).

Adverse events and adverse device effects were not separately collected as part of this study.

### 2.4. Statistics

The intention-to-treat (ITT) population consisted of all users with valid informed consent who had been exposed to the DLNS.

Endpoints, including the OLI tool, WPAI, and time spent with HCPs, were analysed using a linear mixed model. The individual means and the contrasts between baseline and 3 and 6 months, respectively, as well as the 95% confidence intervals, were estimated. Furthermore, it was tested if the contrasts were equal to zero.

Endpoints, including the number of baseplate changes, number of baseplate changes due to leakage worry, use of supporting products, and number of leakages outside the baseplate, were analysed using a negative binomial model. The relative risks between baseline and 3 and 6 months, respectively, were estimated and tested for equality to 1. Furthermore, the mean change in percent with a 95% confidence interval was estimated.

For both types of models, visits (0, 3, and 6 months) were included as a fixed effect. The models took into consideration that measurements from the same individuals at different visits may be correlated. An unstructured correlation matrix was applied.

For all statistical analyses, a 2-sided significance level of 5% was applied. Statistical analyses were performed using SAS v. 9.4 (SAS Institute Inc., Cary, NC, USA). *p* < 0.05 (*), *p* < 0.01 (**), and *p* < 0.001 (***).

### 2.5. Ethical Consideration

The study follows applicable laws and regulations that apply to an observational study in accordance with the Declaration of Helsinki, ISO 14155:2020 [14], and European Medical Device Regulation 2017/745. According to the Health Research Authority in the UK, this study did not require review by an NHS Research Ethics Committee. All participants were fully informed about the study, and all gave informed consent electronically to participate.

### 2.6. Role of the Funding Source

The study was sponsored by Coloplast and was conducted in collaboration with QC Medica. QC Medica was involved in the management of the study questionnaires and the development of the data collection tool. The sponsor was involved in the study design, in the analysis and interpretation of data, in writing the report, and in the decision to submit the paper for publication.

## 3. Results

### 3.1. Demographics of Study Participants

This interim analysis was based on the first n = 100 participants who could have completed the 6-month questionnaire. At the 6-month follow-up, n = 2 participants were lost to follow-up, and n = 3 participants had dropped out of the study. Participants had dropped out of the study due to loss of prescription for the DLNS (n = 1) and unexpected stoma reversal (n = 2), and were therefore not related to DLNS use. At the database lock for the interim analysis, n = 84 participants had completed the 6-month questionnaire, whilst n = 11 participants had not yet completed the questionnaire; however, they may potentially contribute with data later in the study.

Out of the n = 100 participants included in this interim analysis, not all provided answers to all questions in the baseline questionnaire. Demographics and stoma information are provided based on the number of participants responding to each question. The ITT population consisted of 61% females, and the mean age was 50.9 years (Table 1). Participants, on average, had their stoma surgery 7.0 years prior to enrolment in the study. Thirty percent of the participants had been living with their stoma for less than a year, and 70% of the participants had been living with their stoma for a year or more. Eighty-six percent of the participants had an ileostomy, and fourteen percent had a colostomy.

Participants had, on average, used the DLNS together with 6.6 (SD = 3.7; range = 0 to 10) out of the preceding 10 baseplates at 3-month follow-up, and with 5.6 (SD = 4.1; range = 0 to 10) out of the preceding 10 baseplates at 6-month follow-up.

### 3.2. Leakages Outside the Baseplate

**Figure 1:**
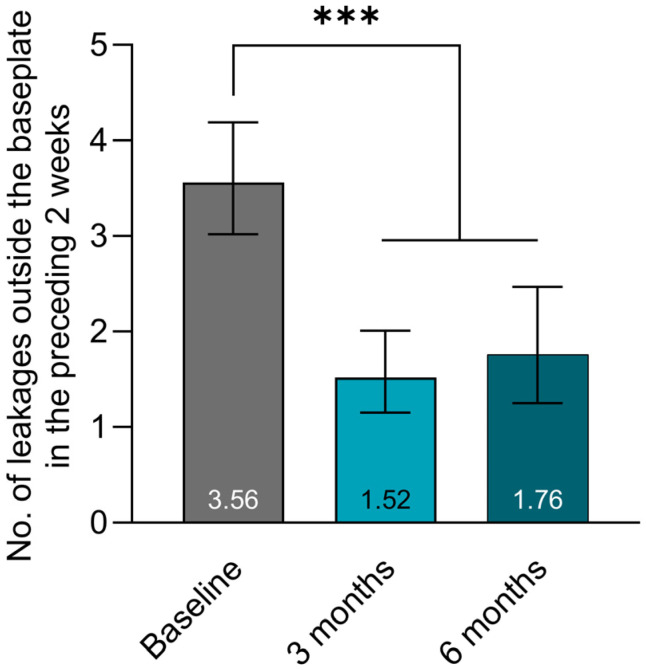
Number (No.) of leakages outside the baseplate for the ITT population. Data are presented as LS means, and error bars represent the corresponding 95% confidence intervals. *** *p* < 0.001.

The LS mean number of leakages outside the baseplate during the preceding 2 weeks was 3.56 (95% CI [3.02; 4.19]) at baseline, which decreased to 1.76 (95% CI [1.25; 2.47]) after usage of the DLNS for 6 months (Figure 1). This corresponds to a 51% (95% CI [31%; 65%] reduction in the number of leakages outside the baseplate (*p* < 0.001). Only a minor difference was observed between the 3- and 6-month follow-up.

### 3.3. Leakage-Related Quality of Life

**Figure 2:**
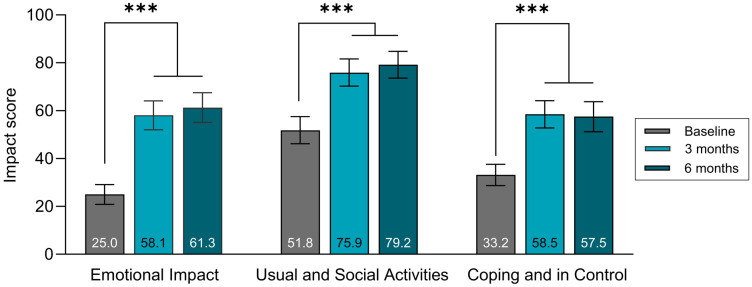
Ostomy Leak Impact domain scores for the ITT population. Data are presented as LS means, and error bars represent the corresponding 95% confidence intervals. *** *p* < 0.001.

All three domains of the OLI significantly improved when using the DLNS for 3- and 6 months compared to baseline (Figure 2). The LS mean Emotional Impact domain score was 25.0 (95% CI [20.9; 29.1]) at baseline, which increased to 61.3 (95% CI [55.1; 67.5]) after usage of the DLNS for 6 months, corresponding to an improvement in the LS mean score of 36.3 points (95% CI [30.2; 42.4]; *p* < 0.001). The LS mean Usual and Social Activities domain score was 51.8 (95% CI [46.2; 57.5]) at baseline, which increased to 79.2 (95% CI [73.6; 84.8]) after usage of the DLNS for 6 months, corresponding to an improvement in the LS mean score of 27.3 points (95% CI [21.9; 32.8]; *p* < 0.001). The LS mean Coping and in Control domain score was 33.2 (95% CI [28.7; 37.6]) at baseline, which increased to 57.5 (95% CI [51.2; 63.8]) after usage of the DLNS for 6 months, corresponding to an improvement in the LS mean score of 24.3 points (95% CI [17.9; 30.7]; *p* < 0.001) (Figure 2).

### 3.4. Use of Baseplates

At baseline, the LS mean number of baseplate changes (or entire system for 1-pc users) was 12.46 (95% CI [11.24; 13.82]) in the preceding 2 weeks (Table 2). After usage of the DLNS for 6 months, the LS mean number of baseplate changes was 10.70 (95% CI [9.44; 12.13]) in the preceding 2 weeks, corresponding to a reduction in LS mean values of 14% (95% CI [6%; 22%]; *p* = 0.002).

The LS mean number of unplanned baseplate changes (or entire system for 1-pc users) due to worry about leakage (not an actual leakage) in the preceding 2 weeks was 8.07 (95% CI [6.90; 9.44]) at baseline, which decreased to 4.26 (95% CI [3.35; 5.42]) after usage of the DLNS for 6 months, corresponding to a mean reduction in the LS mean score of 47% (95% CI [33%; 59%]; *p* < 0.001) (Table 2). This observation was consistent at 3- and 6-month follow-up.

### 3.5. Use of Supporting Products

Between baseline and 6-month follow-up, participants reported significantly decreased use of: paste (tubes) (*p* = 0.044), adhesive tape (pieces) (*p* = 0.001), adhesive remover-wipes (items) (*p* = 0.013), skin cleanser-wipes (items) (*p* = 0.001), and deodorant (bottles) (*p* = 0.020). For the remaining types of supporting products, changes in usage were not statistically significant, but most trended towards lower use (Table 2).

### 3.6. Interactions with HCPs

The LS mean total time spent with HCPs (excluding Coloplast interactions) was 199.2 min (95% CI [150.2; 248.2]) in the preceding 3 months at baseline (Table 3). After usage of the DLNS for 6 months, the LS mean time spent with HCPs (excluding Coloplast interactions) was 69.3 min (95% CI [50.4; 88.1]) in the preceding 3 months, corresponding to a difference in LS mean values of −129.9 min (95% CI [−176.8; −83.0]; *p* < 0.001) compared to baseline. A gradual decrease in total time spent with HCPs was observed over the study period. Lower time spent with SCNs in a clinic had the largest contribution to the reduction in total time spent with HCPs.

### 3.7. Work Productivity and Activity Impairment

Fifty-one percent of the participants were employed at baseline. Minor changes in employment status were observed during the study period from baseline to 6-month follow-up (Table 4). Absenteeism rate (30.7%), Presenteeism rate (53.4%), Productivity loss rate (59.5%), and Activity Impairment (61.8%) were all relatively high at baseline; nonetheless, all four metrics had significantly improved at 6-month follow-up (Table 4).

## 4. Discussion

Multiple positive health-related outcomes were identified when using the DLNS for 6 months, along with benefits related to healthcare resource utilisation and work productivity.

When using the DLNS, participants reported significant improvement in the *Emotional Impact* domain score of the OLI tool compared with baseline (Δ36.3 points), which is much higher than the previously described minimal clinically important difference (MCID) value of Δ7.6 (average of 3 MCID values derived from different methods) [12]. Similar improvements were observed after 3 months of DLNS use, indicating a sustained improvement within the first half year of DLNS use. Based on the items in the domain, this improvement indicates that users felt less frustration, embarrassment, worry, and panic and had better sleep while using the DLNS.

The baseline Emotional Impact domain score of 25.0 is markedly lower than what we have previously observed in the clinical studies with the DLNS [7,8,9], which underscores that the participants in this report struggle to a great extent with leakage-related complications. Indeed, the participants reported a very high number of leakage incidents at baseline, with 3.8 episodes of leakage progressing outside the baseplate in the preceding 2 weeks. In a recent cross-sectional study from the UK [15], participants (n = 301) who experienced leakages outside the baseplate at least once per week had an Emotional Impact score of 38.5, thus supporting that weekly leakages outside the baseplate have significant emotional consequences for the afflicted.

The participants also reported significant improvements in the Usual and Social Activities and Coping and in Control domains from baseline to follow-up after 3 and 6 months. The Usual and Social Activities domain score increased by 27.3 points between baseline and 6-month follow-up, and the Coping and in Control domain score increased by 24.3 points. The observed improvements in the two domains were greater than the respective MCID values of Δ6.6 and Δ7.2 [12]. These results add to the relevance of the DLNS in providing a meaningful improvement for users in how much their QoL is impacted by leakage, and indicate that users overall felt greater engagement in social activities, as well as being better able to cope with and control their situation. These findings corroborate the results of the previous clinical studies with the DLNS [8,9]; nonetheless, it is worth noting that the effect sizes of the OLI domains in the present study are greater than previously observed. The present study supports that the health benefits observed in clinical trials with intervention periods up to 3 months are maintained in the longer term as well.

Besides investigating long-term health benefits associated with DLNS use, the study also assessed implications of its use on healthcare resource utilisation. The participants reported 12.46 changes in baseplates in the preceding 2 weeks at baseline, which significantly decreased to 10.70 changes in baseplates at 6-month follow-up, corresponding to a reduction of 14%. Lower use of baseplates was partly attributed to the secondary endpoint, where participants reported that they had fewer changes in baseplates due to worry about leakage, which may be partly offset by more changes due to leakage notifications. This indicates that participants gained more appropriate changing patterns when using the DLNS. Utilisation of some supporting products also significantly decreased when using the DLNS, such as adhesive tape and paste, which are often used to mitigate the risk of leakage. Reduced utilisation of supporting products may also be a result of fewer baseplate changes.

Our data corroborate previous study findings that people who are burdened by leakages increase their use of baseplates and bags, as well as supporting products to mitigate the risk of future leakage incidents and to cope with the associated worry [6,16]. Excessive use of stoma care products is a major concern for healthcare authorities. Consequently, prescription guidelines have been published in the UK to help alleviate this [17]. Taken together, this data suggests that reducing people’s worry about leakage and the number of leakages outside the baseplate may reduce inappropriate use of baseplates, bags, and supporting products.

People burdened by leakages have also reported interacting more frequently with HCPs to address their leakage-related issues, especially with SCNs [16]. In the present study, participants reported significant reductions in total time spent with HCPs from baseline to 6-month follow-up, mostly driven by a reduction in time spent with SCNs in a clinic. By reducing leakage and the associated worry, the DLNS users might not have reasons to engage with their SCN to receive advice on managing and living with leakage and leakage-related worry. The significant amount of time saved by the SCNs with each DLNS user might enable them to focus resources on patients who continue to struggle with other stoma-related complications. SCNs constitute a valuable but limited resource in healthcare today, and it is now the ambition of the National Health Service (NHS) in the UK to implement digital solutions that can free up the time of HCPs [18].

Stoma formation and the required management and care can also have a negative impact on the ability to work, potentially leading to periods of sickness absence or early retirement, or a change in occupation [6,19,20,21,22,23]. Furthermore, a substantial proportion of people living with a stoma have reported that leakage and the associated worry have negative implications on their ability to work [6]. In the present study, participants in employment reported that they had missed 30.7% of work hours in the preceding 7 days due to health problems at baseline, which decreased significantly to 15.8% at 6-month follow-up, corresponding to a 14.9% difference. The employed participants also reported that at baseline they experienced 53.4% reduced productivity at work due to health problems, which decreased significantly to 37.7% at 6-month follow-up, corresponding to a 15.7% difference. The overall work impairment was 59.5% at baseline, which decreased significantly to 41.9% at 6-month follow-up, corresponding to a 17.6% difference.

Minimal important differences for the individual metrics of the WPAI tool have not been established for people living with a stoma. MCID-values are sensitive to different populations and clinical scenarios; however, for patients with Crohn’s disease, the following MCID values have been reported: 6.5% (absenteeism), 6.1% (presenteeism), 7.3% (overall work impairment), and 8.5% (activity impairment) [24]. These values have also been adopted for patients with ulcerative colitis [25]. The improvements observed across all four metrics were higher than the MCID values established for patients with Crohn’s disease, indicating that the observed differences in all four metrics can be considered relevant.

The observed baseline values of absenteeism, presenteeism, overall work impairment, and activity impairment are relatively high when compared to patients with inflammatory bowel disease (Crohn’s disease and ulcerative colitis) [26,27] or patients performing transanal irrigation for managing neurogenic bowel dysfunctions [28]. Disease severity naturally has major implications for the impairments reported in these studies—e.g., absenteeism was 16.4% for people with mild ulcerative colitis and 49.1% for people with moderate/severe ulcerative colitis in an analysis not adjusting for demographic factors [26]. Overall, this data indicates that living with gastrointestinal complications can have profound negative implications on the ability to work. Thus, use of the DLNS may not only have profound positive implications for the individual but may also provide societal gains when considering improvements to work productivity.

### Limitations

The results of this study should be interpreted considering the limitations of its design. The study was an observational, real-world evidence investigation without a control group. In studies without a control group, it is difficult to discriminate between the effects of (1) the product, (2) natural adaptations and improvements over time, and (3) study effects. A potential study effect may be the Hawthorne effect, where people change behaviour, often improving performance, because they know they are being observed or studied, rather than due to actual changes in experimental conditions. Benefits related to leakage control and improvements to QoL were, however, also observed in a previously conducted randomised-controlled cross-over clinical trial with the DLNS (without the remote support service) with an intervention period of 8 weeks [9]. In order to more robustly validate the true effect of the DLNS on product utilisation, interactions with HCPs, and work productivity, future randomised controlled trials or controlled cohort studies are needed to assess these outcomes. When the full dataset of the study is available, we will be able to compare effect sizes on users with a recently formed stoma and experienced users, which will provide valuable information on whether these groups experience benefits of using the DLNS to a similar degree.

Furthermore, baseline values may be influenced by recall bias, since users were not told to monitor outcomes, such as leakage and healthcare resource utilisation, until they were actively participating in the study. Finally, participants were not instructed to answer the WPAI questionnaire focusing only on stoma-related complications, as the questionnaire has not been validated for this patient group.

## 5. Conclusions

In this interim analysis, participants reported significant reductions in leakage episodes outside the baseplate and great improvements in QoL when using the DLNS together with a remote support service. The baseline values for QoL were very low, and the number of leakages outside the baseplate was higher than previously observed in our clinical studies with the DLNS, indicating that the enrolled participants (being part of this interim analysis) were struggling to a great extent. The full analysis set at study completion will reveal whether the participants involved in the interim analysis formed a particularly struggling population and whether the clinical benefits and improvements on healthcare resource utilisation are maintained throughout the entire study period of one year.

Participants reported significantly less time spent with different types of HCPs in relation to their stoma care. Additionally, use of the DLNS was associated with reductions in the number of baseplate changes and a decreased usage of some supporting products, which may help ensure more appropriate use of stoma care products and overall healthcare resource utilisation. Participants in employment reported less time missed at work due to health problems and higher productivity when using the DLNS, thus providing potential gains for society.

## Figures and Tables

**Table 1:** Demographics and stoma information for the ITT population.

	ITT Population
Age in years; Mean ± SD (range); (n = 98)	50.9 ± 15.8 (19; 84)
Sex; females/males; n (%); (n = 98)	60 (61.2%)/38 (38.8%)
Time since stoma formation in years; Mean ± SD (range); n = 97	7.0 ± 12.8 (0; 74)
New patients discharged (<1 year with a stoma); n (%)	29 (29.9%)
Experienced users (≥1 year with a stoma); n (%)	68 (70.1%)
Reason for stoma surgery; n (%); (n = 97)	
Crohn’s disease	15 (15.5%)
Ulcerative colitis	26 (26.8%)
Cancer	25 (25.8%)
Other	31 (32.0%)
Type of stoma; ileostomy/colostomy; n (%); (n = 97)	83 (85.6%)/14 (14.4%)
Ostomy solution brand; n (%); (n = 97)	
Coloplast	70 (72.2%)
Another manufacturer	27 (27.8%)
Ostomy solution; n (%); (n = 97)	
1-piece	79 (81.4%)
2-piece	14 (14.4%)
Do not know	4 (4.1%)
Baseplate type; n (%); (n = 97)	
Flat	14 (14.4%)
Convex (soft)	46 (47.4%)
Convex (deep)	24 (24.7%)
Concave	8 (8.2%)
Do not know	5 (5.2%)

**Table 2:** Number of baseplates and supporting products used in the preceding 2 weeks at baseline, 3-month and 6-month follow-up for the ITT population. Significant changes are marked with bold font.

	Baseline LS Mean (95% CI)	3 Months LS Mean (95% CI)	6 Months LS Mean (95% CI)	Effect Size (Baseline vs. 6 Months) Percent Reduction (95% CI)	*p*-Value
Baseplate changes	12.46 (11.24; 13.82)	10.35 (9.22; 11.62)	10.70 (9.44; 12.13)	14% (6%; 22%)	***p* = 0.002**
Unplanned baseplate changes	8.07 (6.90; 9.44)	3.98 (3.08; 5.15)	4.26 (3.35; 5.42)	47% (33%; 59%)	***p* < 0.001**
Rings/seals	8.17 (6.65; 10.03)	6.79 (5.46; 8.45)	6.83 (5.62; 8.29)	16% (−0%; 30%)	*p* = 0.052
Paste (tubes)	0.89 (0.46; 1.72)	0.37 (0.19; 0.76)	0.35 (0.17; 0.72)	60% (3%; 84%)	***p* = 0.044**
Paste (strips)	0.82 (0.33; 2.05)	0.59 (0.22; 1.60)	0.35 (0.09; 1.34)	58% (−112%; 92%)	*p* = 0.295
Adhesive tape (pieces)	8.82 (6.92; 11.24)	5.53 (4.03; 7.59)	6.08 (4.51; 8.20)	31% (14%; 45%)	***p* = 0.001**
Adhesive remover-spray (bottles)	3.29 (2.56; 4.22)	2.61 (2.08; 3.29)	2.91 (2.18; 3.89)	11% (−23%; 36%)	*p* = 0.468
Adhesive remover-wipes (Items)	4.65 (3.33; 6.50)	3.09 (2.17; 4.41)	2.89 (2.01; 4.14)	38% (10%; 57%)	***p* = 0.013**
Skin cleanser wipes (items)	11.39 (9.22; 14.07)	9.28 (7.48; 11.53)	7.60 (5.82; 9.94)	33% (14%; 48%)	***p* = 0.001**
Skin barrier spray (bottles)	0.79 (0.43; 1.45)	0.53 (0.30; 0.96)	0.60 (0.37; 1.00)	24% (−42%; 59%)	*p* = 0.398
Skin barrier wipe (items)	4.26 (3.00; 6.04)	2.75 (1.87; 4.05)	3.68 (2.47; 5.50)	14% (−22%; 39%)	*p* = 0.406
Skin barrier cream (bottles)	0.31 (0.11; 0.87)	0.11 (0.05; 0.25)	0.11 (0.04; 0.29)	64% (−26%; 90%)	*p* = 0.110
Skin barrier sheets (items)	1.15 (0.49; 2.70)	0.93 (0.35; 2.45)	0.69 (0.24; 1.99)	40% (−62%; 78%)	*p* = 0.314
Belts	1.53 (0.96; 2.46)	1.34 (0.93; 1.93)	1.71 (1.14; 2.57)	−12% (Increase) (−68%; 26%)	*p* = 0.595
Powder (bottles)	1.21 (0.79; 1.87)	0.88 (0.58; 1.33)	0.84 (0.50; 1.41)	31% (−0%; 53%)	*p* = 0.051
Deodorant (sachets)	1.86 (0.94; 3.69)	2.16 (1.19; 3.94)	2.45 (1.32; 4.54)	−31% (Increase) (−87%; 8%)	*p* = 0.128
Deodorant (bottles)	0.62 (0.32; 1.21)	0.27 (0.13; 0.57)	0.24 (0.14; 0.41)	61% (14%; 82%)	***p* = 0.020**

**Table 3:** Time spent in minutes with different HCPs and Coloplast stoma support in the preceding 3 months at baseline, 3-month, and 6-month follow-up for the ITT population. Significant changes are marked with bold font.

	Baseline LS Mean (95% CI)	3 Months LS Mean (95% CI)	6 Months LS Mean (95% CI)	Effect Size (Baseline vs. 6 Months) LS Mean Difference (95% CI)	*p*-Value
SCN in a clinic	73.7 (53.9; 93.5)	36.1 (23.7; 48.5)	17.1 (9.6; 24.7)	−56.5 (−76.5; −36.6)	***p* < 0.001**
SCN at home	17.1 (7.9; 26.3)	6.1 (2.1; 10.0)	1.6 (−0.1; 3.4)	−15.4 (−24.4; −6.4)	***p* = 0.001**
Nurse (non-face-to-face, e.g., phone call) ^1^	33.3 (21.4; 45.2)	12.6 (5.9; 19.3)	12.8 (4.0; 21.6)	−20.5 (−35.4; −5.6)	***p* = 0.008**
General practitioner (in person) ^1^	22.4 (10.0; 34.8)	11.5 (6.3; 16.7)	11.0 (5.4; 16.6)	−11.4 (−23.9; 1.1)	*p* = 0.072
General practitioner (non-face-to-face, e.g., phone call) ^1^	12.3 (6.9; 17.8)	6.8 (2.7; 11.0)	4.6 (1.8; 7.5)	−7.7 (−13.6; −1.8)	***p* = 0.011**
Dermatologist ^1^	2.1 (−0.6; 4.9)	0.9 (−0.5; 2.3)	1.2 (−0.2; 2.7)	−0.9 (−4.1; 2.3)	*p* = 0.571
Gastroenterologist ^1^	13.8 (5.7; 21.8)	9.8 (5.0; 14.6)	10.7 (5.3; 16.1)	−3.1 (−8.7; 2.5)	*p* = 0.281
Stoma surgeon ^1^	20.9 (9.7; 32.2)	7.6 (4.4; 10.7)	10.0 (5.4; 14.5)	−11.0 (−22.5; 0.6)	*p* = 0.063
Home care nurse ^1,2^	3.1	0.0	0.4	-	-
Coloplast stoma support (Charter and/or Heylo™ Support Service)	33.4 (24.9; 41.9)	29.2 (18.9; 39.6)	9.7 (5.9; 13.5)	−23.7 (−33.3; −14.2)	***p* < 0.001**
Total time spent with healthcare professionals (excluding Coloplast interactions)	199.2 (150.2; 248.2)	91.5 (70.6; 112.4)	69.3 (50.4; 88.1)	−129.9 (−176.8; −83.0)	***p* < 0.001**

^1^ Related to stoma care. ^2^ The statistical model did not converge; data provided as arithmetic means.

**Table 4:** Comparison of WPAI scores at baseline, 3-month follow-up, and 6-month follow-up for the ITT population. WPAI: Work Productivity and Activity Impairment Questionnaire. WPAI 1: Absenteeism (the percentage of work time missed due to health problems). WPAI 2: Presenteeism (the percentage of impairment while working due to a health problem). WPAI 3: Productivity loss rate (the percentage of overall work impairment due to health problems, considering both absenteeism and presenteeism). WPAI 4: Percent activity impairment due to health problem (the percentage of daily activities affected by health problems).

	Baseline LS Mean (95% CI)	3 Months LS Mean (95% CI)	6 Months LS Mean (95% CI)	Effect Size (Baseline vs. 6 Months) LS Mean Difference (95% CI)	*p*-Value
Proportion of participants being employed	51 out of 100 (51.0%)	46 out of 95 (48.4%)	37 out of 84 (44.0%)	-	-
WPAI 1: Absenteeism	30.7% (19.2%; 42.3%)	19.8% (9.6%; 30.1%)	15.8% (5.8%; 25.8%)	−14.9% (−28.8%; −1.0%)	*p* = 0.036
WPAI 2: Presenteeism	53.4% (43.7%; 63.1%)	40.8% (31.2%; 50.4%)	37.7% (26.9%; 48.5%)	−15.7% (−26.5%; −4.9%)	*p* = 0.006
WPAI 3: Productivity loss rate	59.5% (49.7%; 69.3%)	47.8% (37.8%; 57.9%)	41.9% (30.6%; 53.2%)	−17.6% (−28.8%; −6.5%)	*p* = 0.003
WPAI 4: Activity impairment	61.8% (55.8%; 67.8%)	48.8% (42.6%; 55.0%)	45.4% (38.9%; 51.8%)	−16.4% (−22.3%; −10.6%)	*p* < 0.001

## Data Availability

Anonymous data and study protocol are available from the corresponding author on reasonable request.
